# Advances in the Epidemiological Surveillance of Tick-Borne Pathogens

**DOI:** 10.3390/pathogens12050633

**Published:** 2023-04-23

**Authors:** Adrian Alberto Díaz-Sánchez, Dasiel Obregón, Huarrisson Azevedo Santos, Belkis Corona-González

**Affiliations:** 1 Department of Biology, University of Saskatchewan, 112 Science Place, Saskatoon, SK S7N 5E2, Canada; adiasanz88@gmail.com; 2 School of Environmental Sciences, University of Guelph, Guelph, ON N1G 2W1, Canada; dasieloa@uoguelph.ca; 3 Department of Epidemiology and Public Health, Veterinary Institute, Federal Rural University of Rio de Janeiro, Seropédica 23890-000, RJ, Brazil; huarrisson@yahoo.com.br; 4 Department of Animal Health, National Center for Animal and Plant Health, San José de las Lajas CP 32700, Mayabeque, Cuba

Ticks are obligate blood-feeding ectoparasites of mammals, birds, and reptiles, which are globally important vectors of pathogens that impact both human and animal health [1]. Ticks are a major group of arthropod vectors second only to mosquitoes in importance as vectors species; they harbor and transmit a wider variety of pathogens than any other blood-sucking arthropod, including bacteria, protozoa, viruses, and helminths [2]. The number of known tick-borne pathogens has increased dramatically since the 1980s, hence this problem is becoming increasingly urgent due to its socioeconomic impact, mainly related to reduced livestock production in countries of the Southern Hemisphere [3]. The spread of tick-borne diseases is increasing due to global climate change, land-use change, human population growth, translocation, and movement of animals [4,5]. The current climate-driven redistribution of ticks and other hematophagous arthropods poses a challenge to public health and veterinary services, due to the emergence and re-emergence of pathogens that threaten humans and animals [6].

The successful prevention and control of tick-borne diseases depend on accurate knowledge of the prevalence and the risk factors associated with pathogen transmission and host infection [7]. Particularly, early diagnosis and management are essential for the control of tick-borne diseases, both at individual and population levels, mainly due to symptoms of these diseases being nonspecific, which can make diagnosis challenging [8]. Researchers in this field are doing great work at understanding the practical aspects of epidemiology, clinical assessment, treatment, laboratory diagnosis, surveillance, and prevention of tick-borne diseases. For instance, the Special Issue titled “Advances in the Epidemiological Surveillance of Tick-Borne Pathogens” assembles a series of eight selected papers (i.e., one review paper and seven research papers), that attempt to address various fundamental components of tick-borne diseases from different regions around the world.

The One Health approach is currently being proposed as the foundation for the study of pathogens that affect animal health, considering the interconnections between human, animal, and environmental health. This approach incorporates a multidisciplinary perspective, involving different fields such as genomics, epidemiology, pathogenesis, and virulence factor studies that lead to the development of new tools and technologies for detecting and monitoring pathogens in different settings [9]. In this Special Issue, Dantán-González, et al. [10] describes the first comparative genomics and phylogeographic analyses of seven draft genomes of Mexican strains of *Anaplasma marginale*, the etiologic agent of bovine anaplasmosis. A total of 24 genomes of *A. marginale* retrieved from the GenBank database were characterized in this study, where genetic diversity and phylogeographic analyses were performed using both *msp1*a gene and 5′-UTR microsatellite sequences, as well as the construction of a phylogenetic tree with 540 concatenated genes from the core genome. Interestingly, the Mexican strains of *A. marginale* contained 15 different tandem repeat (TR) sequences in six MSP1a and phylogeographic relationships with strains from North America, South America, and Asia, which confirms their considerable genetic diversity. Pan-genomic analysis between the seven Mexican strains revealed that there are genes that are only present in the local strains.

Bovine babesiosis is one of the most important infectious diseases due to its major economic impact on the cattle industry worldwide, mostly in terms of mortality and morbidity of livestock animals. The global distribution of this disease is dependent on the cattle fever ticks *Rhipicephalus (Boophilus) microplus* and *Rhipicephalus annulatus*, which are responsible for the transmission of *Babesia bovis* and *Babesia bigemina*, the causative agents of bovine babesiosis [11]. To date, since there is not an effective vaccine commercially available, the management strategies against bovine babesiosis have been mostly based on surveillance programs through the diagnosis, treatment, and prevention of not only tick infestations but also the pathogens that they transmit [12]. In *B. bigemina*, the 45 kilodaltons glycoprotein GP-45 is exposed on the surface of the merozoite, it is characterized by a high genetic and antigenic polymorphism, and it is believed to play a role in the invasion of erythrocytes. Initially, GP-45 was proposed as a potential vaccine candidate, since experimental evidence demonstrated that cattle vaccinated with a purified native GP-45 generated partial protection, with a significantly lower percentage of parasitemia, compared with unvaccinated cattle when challenged with a *B. bigemina* virulent strain [13]. The paper from Mercado-Uriostegui, et al. [14] in this article collection described that the GP-45 protein contains conserved B-cell epitopes that were found in geographically distant isolates of *B. bigemina*, which induce neutralizing antibodies that block the invasion process to erythrocytes in vitro, suggesting that this protein plays a critical role in the survival of the parasite under field conditions. Accordingly, the conserved GP-45 peptides found in this study could be used to develop new diagnostic methods and vaccine candidates.

In this special issue, another original paper was published by Beristain-Ruiz, et al. [15], who reported the association between the prevalence of several tick-borne pathogens and the infestation by *Rhipicephalus sanguineus* sensu lato in dogs from Juarez City, in the Northwest Mexico—United States border. In this work, *R. sanguineus* s.l. was identified as the main tick species naturally parasitizing the sampled dogs. The presence of tick-borne pathogens such as *Ehrlichia canis*, *Anaplasma platys*, *Rickettsia rickettsii* was detected in both ticks and dog blood samples, while *Anaplasma phagocytophilum* was found only in dog blood samples. Notably, these findings represent the first molecular evidence of *R. rickettsii* infections in domestic dogs from Mexico. On the other hand, the study published by Georges, et al. [16] described the comparison of different diagnostic methods, including the microscopic examination of peripheral blood smears, autologous cell cultures, and reverse line blot hybridization, used for the screening of *Anaplasma*/*Ehrlichia* sp. in both roaming and symptomatic dogs from Trinidad. The results showed an acceptable concordance between the autologous cell cultures and reverse line blot hybridization, which suggests that both diagnostic methods can be used to detect *E. canis* in subclinical and clinical cases of canine ehrlichiosis.

Equine piroplasmosis is the most common tick-borne disease of Equids, affecting horses, donkeys, mules, and zebras [17]. The etiological agents of the disease around the world are the piroplasmids *Babesia caballi* [18], *Theileria equi* [19], and *Theileria haneyi* [20]. This disease is a severe issue for the horse industry and its control is crucial for the international trade of horses. Experimental studies have demonstrated that *R.* (*Boophilus*) *microplus* acts as a biological vector of *T. equi* in both transstadial and intrastadial transmission modes [21]. In this Special Issue, Peckle, et al. [22] published a study aimed at evaluating the dynamics of *T. equi* infection in *R.* (*B.*) *microplus* fed on a chronically infected horse through the infection rate and parasitic load in both pooled tick and ixodid organs samples, at different stages of tick life cycle during the experimental infestation. The experimental findings indicated a strong possibility of transstadial transmission of *T. equi* in *R.* (*B.*) *microplus*, while the adult male tick may play a role in the intrastadial transmission to naïve horses. Interestingly, this study shows high infection rates and a parasitic load of *T. equi* infection in *R.* (*B.*) *microplus* that increased over the experimental period, which confirms the importance of *T. equi* chronically infected horses as a source of infection for *R.* (*B.*) *microplus.* In another way, Torres, et al. [23] investigated the serological and molecular occurrence of *Babesia caballi* and *Theileria equi*, as well as their genetic diversity in thoroughbred horses from racecourses in Chile. The phylogenetic and haplotype analyses were performed using the *B. caballi* 48 KDa rhoptry protein (RAP-1) and *T. equi* 18S rRNA genes sequences, and the results revealed a low genetic diversity among the Chilean strains, which may have come from those reported in Brazil, Israel, or Cuba. This is the first report of *B. caballi* and *T. equi* infections in Chilean thoroughbred racing horses.

Tick-borne rickettsial pathogens are amongst the emerging and re-emerging zoonoses of public health importance since continue causing severe illness and death in either healthy adults or children, despite the availability of effective antibacterial therapy [8]. Traditionally rickettsiae members have been considered to be transmitted by ixodid ticks, while the role of argasid ticks as competent vectors of these pathogens remains still unexplored. Chitanga, et al. [24] investigated the presence of *Rickettsia* spp. in soft tick species *Ornithodoros moubata* and *Ornithodoros porcinus* collected from warthog burrows in two national parks of Zambia. In this study, the presence of *Rickettsia lusitaniae* in *O. porcinus* tick species was confirmed by DNA nucleotide sequence and phylogenetic analyses based on rickettsial citrate synthase (*glt*A) and 17kDa common antigen (*htr*A) genes. This discovery adds to our understanding of the geographic distribution and vector range of rickettsial pathogens in sub-Saharan Africa.

Lyme borreliosis is another zoonotic disease caused by some genospecies of the spirochete *Borrelia burgdorferi* sensu lato (s.l.) complex, which is the most prevalent tick-borne disease in humans in the northern hemisphere. In this Special Issue, Ji, et al. [25] published a systematic review and meta-analysis of the prevalence of *B. burgdorferi* in Ixodidae ticks around Asia. The literature analysis included 91 articles published until March 2021, and the meta-analysis identified data from nine countries including China, Japan, Malaysia, Mongolia, Pakistan, Russia Siberia region, South Korea, Thailand, and Turkey, as well as tick species from six genera, namely *Amblyomma*, *Dermacentor*, *Haemaphysalis*, *Hyalomma*, *Ixodes*, and *Rhipicephalus* screened for infection with *B. burgdorferi.* According to the findings of this study, ticks of the genera *Ixodes*, *Haemaphysalis,* and *Dermacentor* are the most frequent *B. burgdorferi* vectors in Asia. Despite the literature focused mostly on East Asia, and although data are still scarce, this meta-analysis is the first attempt to explain the *B. burgdorferi* infection in Asian ixodid ticks, and it serves as a reference for future research.

In conclusion, this Special Issue contains a cutting-edge collection of papers on tick-borne diseases from throughout the world, focusing on ecoepidemiology, surveillance, diagnosis, management of risks, and prevention of TBDs. The published results provide insights with important implications for other researchers in academia, government, and industry working to develop successful strategies for preventing and controlling vector ticks and the pathogens they transmit.
